# Hypoallergen Peanut Lines Identified Through Large-Scale Phenotyping of Global Diversity Panel: Providing Hope Toward Addressing One of the Major Global Food Safety Concerns

**DOI:** 10.3389/fgene.2019.01177

**Published:** 2019-11-27

**Authors:** Arun K. Pandey, Hari K. Sudini, Hari D. Upadhyaya, Rajeev K. Varshney, Manish K. Pandey

**Affiliations:** International Crops Research Institute for the Semi-Arid Tropics (ICRISAT), Hyderabad, India

**Keywords:** reference set, peanut allergens, Ara h 1, Ara h 2, Ara h 3, Ara h 6, Ara h 8, enzyme-linked immunosorbent assay

## Abstract

Peanut allergy is one of the serious health concern and affects more than 1% of the world’s population mainly in Americas, Australia, and Europe. Peanut allergy is sometimes life-threatening and adversely affect the life quality of allergic individuals and their families. Consumption of hypoallergen peanuts is the best solution, however, not much effort has been made in this direction for identifying or developing hypoallergen peanut varieties. A highly diverse peanut germplasm panel was phenotyped using a recently developed monoclonal antibody-based ELISA protocol to quantify five major allergens. Results revealed a wide phenotypic variation for all the five allergens studied *i.e.*, Ara h 1 (4–36,833 µg/g), Ara h 2 (41–77,041 µg/g), Ara h 3 (22–106,765 µg/g), Ara h 6 (829–103,892 µg/g), and Ara h 8 (0.01–70.12 µg/g). The hypoallergen peanut genotypes with low levels of allergen proteins for Ara h 1 (4 µg/g), Ara h 2 (41 µg/g), Ara h 3 (22 µg/g), Ara h 6 (829 µg/g), and Ara h 8 (0.01 µg/g) have paved the way for their use in breeding and genomics studies. In addition, these hypoallergen peanut genotypes are available for use in cultivation and industry, thus opened up new vistas for fighting against peanut allergy problem across the world.

## Introduction

Food allergy causes severe health issues throughout the globe and the incidences are increasingly recorded across the globe. Even though, approximately 5% of young kids and 4% of adult in western countries are affected by food allergens (Sicherer et al., 2010), the problem has now become more common in developing countries (Liew et al., 2013; Leung et al., 2018). About 40% of the food allergies occur due to the consumption of plants and plant-derived products. Peanut is identified as one of the major sources of food allergy in addition to milk, egg, dry fruits (almonds, cashews, hazelnuts, pistachios, pecans, and walnuts), fish, shellfish, soy, and wheat, with ∼90% cumulative contribution among food allergies in human (Hefle et al., 1996). A large number of population across the world are affected by peanut allergy and several reports are coming more frequently. For instance, the 1% population of Canadian children are allergic to peanuts (Ben-Shoshan et al., 2009) while the prevalence of peanut-based allergy in France and Denmark ranged between 0.3-0.75% and 0.2-0.4%, respectively (Morisset et al., 2005; Osterballe et al., 2005). About 3% of Australians are allergic to peanuts and peanut-based products (Sicherer and Sampson, 2007; Sicherer and Sampson, 2014). It is a big problem in the United Kingdom (UK) as well, and the prevalence of sensitization increased from 1.3 to 3.2% in 3 years old kids (Grundy et al., 2002). Importantly, the prevalence of peanut-based allergy in United States of America (USA) has been increased from 0.4 to 1.4% between 1997 to 2008 (Sicherer et al., 2010). Similarly, peanut allergy has also been reported in the Asian countries such as Singapore and Philippines where 0.47 and 0.43% school children, respectively, were found allergic to peanuts (Shek et al., 2010). Although information from China is not available, however, the situation in China may be similar to Singapore as 76.8% of Singapore residents are Chinese in origin (http://www.singstat.gov.sg/). Although there may be several cases of food allergy in India (Mahesh et al., 2016), however, not much information is available from India on peanut allergy. Keeping in mind the interdependence and trade among countries for producing raw material, processing, and consumption of peanut, such health-concerning features of the crop reduces its importance in international trade and commerce (Pandey et al., 2012; Pandey and Varshney 2018; Varshney et al., 2018;Varshney et al., 2019). Therefore, the countries producing the peanuts and peanut based product with the most safe, nutritious, and healthy features will get a competitive advantage over other producing countries.

All the major food allergies, including peanut, may induce anaphylaxis leading to life-threatening reactions (Dodo et al., 2005; Sicherer and Sampson, 2010) and it is almost impossible to avoid accidental ingestion of peanut-based products (Berger and Smith, 1998; Kagan et al., 2003). Remarkably, food-based allergies cause around 150–200 deaths per year (http://www.startribune.com/peanut-allergy-kills-22-year-old-twin-cities-man/366152021/), largely due to the consumption of peanuts (50–62%) and tree nuts (15–30%) in USA (Lanser et al., 2015). Proteins are the major cause of food allergy, and these proteins are usually highly resistant to heat and proteolysis (Cabanillas et al., 2012). Peanut is the largest source of the immunoglobulin E (IgE)-mediated food allergies and there is no effective treatment due to which the allergic person is forced to avoid consuming peanut or peanut-based products (Wen et al., 2007). However, the peanut being a common food ingredient in many food preparations, it is very challenging for the allergic person to know the composition of these preparations to avoid consumption (Maleki et al., 2000). The threshold of allergen levels differ among the allergic population and even a minute dose of 100 µg of Ara h 1 can trigger an allergic reaction (Warner, 1999). The diagnosis of peanut allergy can be done using different methods such as double-blind, placebo-controlled food challenge (DBPCFC), the basophil activation test, the specific skin prick test (SPT), and the measurement of specific IgE (Hamilton et al., 2010; Lieberman and Sicherer, 2011; Nicolaou et al., 2011).

Of the 32 different types of proteins present in peanut seeds (Pele, 2010), 18 of these proteins show the allergic property (Iqbal et al., 2016). Further, out of 18 peanut allergen proteins mainly Ara h 1, Ara h 2, Ara h 3, and Ara h 6 are considered as major allergens due to their life-threatening reactions recognized by the IgE leading to anaphylaxis (Krause et al., 2010). The remaining allergen proteins are considered as minor allergens as they don’t cause life-threatening allergic reactions (anaphylaxis). Nevertheless, if a person is already sensitive to Bet v 1 allergen caused due to birch pollen, then one of these minor peanut allergens, Ara h 8, shows cross-reactivity with IgE antibodies causing oral allergy syndrome (OAS) (Mittag et al., 2004; Riecken et al., 2008; Kondo and Urisu, 2009). Allergic protein belongs to different protein families namely cupin (vicilin-type, 7S globulin, legumin-type, 11S globulin, glycinin), conglutin (2S albumin), profilin, nonspecific lipid-transfer protein 1, pathogenesis-related protein (PR-10) 14 kDa, oleosin (16 kDa), and seed storage proteins particularly Ara h 1, Ara h 2, Ara h 3, and Ara h 6 (Pele, 2010). Many studies have shown that the most abundant peanut-based allergens (Ara h 1 and Ara h 3 but, Ara h 2 and Ara h 6) bind strongly with peanut allergic IgE and release basophils mediators, which were confirmed in vitro (de Jong et al., 1998; Koppelman et al., 2005; Palmer et al., 2005; Porterﬁeld et al., 2009) and in vivo (Koppelman et al., 2003; Koppelman et al., 2005; Peeters et al., 2007) with regards to food allergy (Porterﬁeld et al., 2009). Although all the five peanut allergens (Ara h 1 and Ara h 3 but, Ara h 2 and Ara h 6) show IgE reactivity to these peanut allergens, however, the Ara h 2 and Ara h 6 allergens are more commonly recognized in children (Flinterman et al., 2007).

Possible solutions to peanut allergy include the development of vaccine or development of allergen-free peanut varieties. Much research has been focussed on diagnosis and cure to minimize the impact of allergens in the human population, however, reducing allergen proteins in peanut varieties and their products can be the best solution. Unfortunately, insufficient scientific information on a total number of allergen genes in the peanut genome and level of phenotypic variability in existing peanut germplasm hinders further research in this area. Therefore, the main objective of this research was to identify hypoallergen peanut lines by screening a large number of diverse germplasm in the peanut reference set (Upadhyaya, 2009). The hypoallergen peanut varieties that have been identified will promote their commercialization and use in the peanut-based industry. These lines and information generated out of this work can be of great importance in efforts toward fighting peanut allergy and ensuring food safety across the world.

## Materials and Methods

### Plant Materials

**Figure 1:**
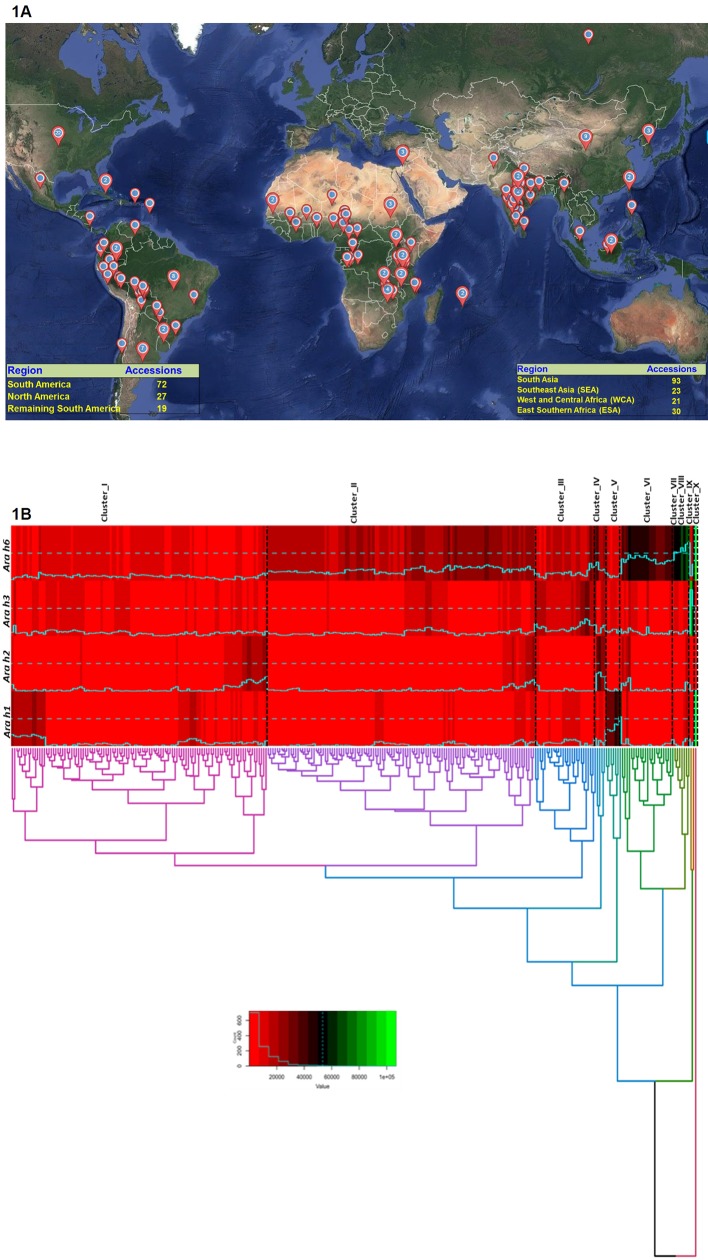
Graphical representation showing global coverage of peanut “reference set” across 51 countries. **(1A)** The world map was constructed by using Maptive software (http://fortress.maptive.com). Out of 300 accessions of the ICRISAT peanut reference set, 93 belongs to South Asia, 23 from Southeast Asia, 21 from West and Central Africa 30 from East and Southern Africa, 72 from South America, 27 from North America, and 19 from remaining South America. **(1B)** Hierarchical clustering on the basis of phenotyping data grouped 300 accessions in 10 cluster. Cluster 1 having 112 accessions, cluster 2 having 117 accessions while cluster 3, and 6 having 27 and 23 accessions respectively. Cluster 7, 8, 9, and 10 showing a high amount of Ara h 6 in their germplasm lines. In cluster 1 all the accessions having a lower amount of allergen content.

The peanut “reference set” consisting of 300 diverse accessions representing 51 countries (Upadhyaya, 2009) (**Supplementary Table 1** and **Figure 1A**) were selected from the composite collection. The reference set included the 184 accessions of the peanut mini core collection (Upadhyaya et al., 2002) that represented diversity of the core collection (1,704 accessions) which in turn represents the diversity of entire International Crops Research Institute for the Semi-Arid Tropics (ICRISAT) peanut genebank collection (14,310 accessions) (Upadhyaya et al., 2003). This set comprised of 264 cultivated species (*Arachis hypogaea*) and 36 wild species and has fair representation for its two subspecies namely *fastigiata* (154) and *hypogaea* (95). The subspecies *fastigiata* was further classified into four botanical varieties namely Fastigiata (70 accessions), Peruviana (5 accessions), Vulgaris (78 accessions), and Aequitoriana (1 accession). Similarly, the other subspecies *hypogaea* was classified into two botanical varieties namely Hirsuta (2 accessions) and Hypogaea (93 accessions) (Holbrook and Stalker 2003). These cultivated genotypes can also be classified into four agronomic types based on their growth habit namely Spanish bunch (73), Valencia bunch (70), Virginia bunch (51), and Virginia runner (33). The seeds for cultivated genotypes (264) were collected from two seasons (Rainy 2016 and Post-rainy 2016–17) for estimating allergen content. Since many of the wild accessions have annual growth period, seeds from two different lots were taken for allergen estimation.

### Sample Preparation and Protein Isolation

Sample preparation and protein isolation were performed following the protocol mentioned in (Pandey et al., 2019). In brief, 2 g of seeds were grinded to make a fine powder and then dissolved in 40 ml of PBS-T (0.05% Tween in phosphate buffered saline, pH 7.4) containing 1 M NaCl in 50 ml falcon tubes (Sarstedt No: 55.476). After 2 h of gentle stirring at room temperature on the rocking platform, the aqueous phase was collected by centrifugation at 2,500 rpm at 4°C for 20 min. The aqueous phase was subsequently centrifuged to remove residual traces and insoluble particles at 3,500 rpm for 10 min at room temperature. Until use, extracts of proteins were stored at −20°C.

### Allergens Estimation Using Enzyme-Linked Immunosorbent Assay

Sandwich format ELISA was used in the study. The peanut allergen proteins were first sandwiched between two antibodies, and then streptavidin-peroxidase was captured. Each peanut sample contains a different quantity of allergen proteins which makes very difficult to estimate the accurate amount of allergen proteins present in seed samples. Dilution is a vital step for ELISA experiment which in turn determines the values of detection range for antibody and target antigen concentrations.

The estimation of peanut allergen through sandwich ELISA were performed according to the recently published protocol (Pandey et al., 2019). Each allergen protein was estimated at different dilution factors (DF). We used a number of dilutions in the peanut samples to detect the specific allergic protein in seeds. The Ara h 1 was detected on three serial doubling dilutions, 1:1,000, 1:2,000, and 1: 4,000 while Ara h 2 and Ara h 3 detected on same dilution 1: 5,000, 1:10,000, and 1: 20,000. In peanut seeds, the Ara h 6 was detected in the high range (1: 40,000, 1: 80,000, and 1:160,000) DF while Ara h 8 detected in a low range of dilution, i.e., 1:10, 1:20, and 1:40.

### Cluster and Data Analysis on the Basis of Allergen Content

Statistical analysis was performed to identify the wide variation of peanut allergens among samples using SigmaPlot (http://www.sigmaplot.co.uk/products/sigmaplot/sigmaplot-details.php). Hierarchical clustering was done using average allergens content of five major allergens (Ara h 1, Ara h 2, Ara h 3, Ara h 6, and Ara h 8) on the basis of similarity matrix generated using HCA (hierarchical cluster analysis). Dendextend statistical package (Galili, 2015) was used for clustering the genotypes on the basis of similarity of average allergen content. This software provides a set of functions for cluster analysis and construction of dendo-gram. The heat map was generated using R package gplots (Warnes et al., 2016) for allergen content of 300 samples.

## Results

### Phenotypic Variation for Allergens Between Cultivated and Wild Gene Pool

**Figure 2:**
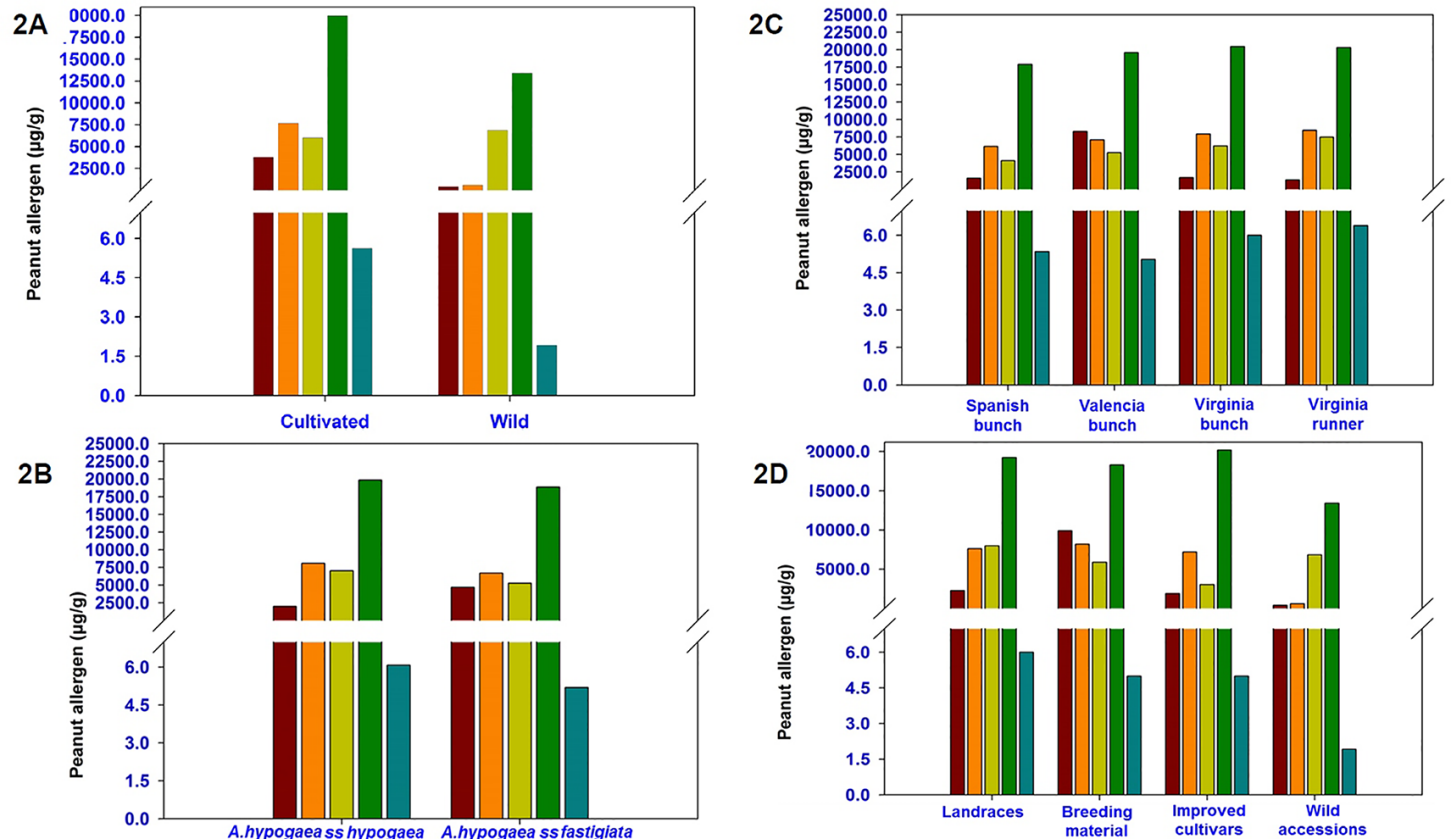
Phenotypic variation for five major peanut allergens **(2A)** between cultivated and wild germplasm accessions **(2B)** between subspecies *hypogaea* and *fastigiata* **(2C)** among different agronomic types **(2D)** and among the biological types of peanut reference set collection.

Phenotyping of 300 diverse accessions (264 cultivated and 36 wild accessions) showed wide phenotypic variations for all the five allergens. Among cultivated accessions, the phenotypic variation was very high for all five major allergens namely Ara h 1 (4–36,833 µg/g), Ara h 2 (52–77,042 µg/g), Ara h 3 (22–106,765 µg/g), Ara h 6 (829–103,892 µg/g), and Ara h 8 (0.01–70 µg/g) as compared to the wild relatives i.e., Ara h 1 (28–1,293 µg/g), Ara h 2 (516–33,055 µg/g), Ara h 3 (1,185–20,474 µg/g), Ara h 6 (4,381–56,858 µg/g), and Ara h 8 (0.3–11 µg/g) (**Table 1**). Except Ara h 3 (6,857 µg/g), the average content of Ara h 1 (404 µg/g), Ara h 2 (606 µg/g), Ara h 6 (13,420 µg/g), and Ara h 8 (2 µg/g) was found lower in wild accessions compared to cultivated genotypes (**Table 1** and **Figure 2A**).

**Table 1:** Range and mean value for five major peanut allergens in peanut germplasm lines of the reference set in the rainy 2016 and 2016–17 postrainy seasons, International Crops Research Institute for the Semi-Arid Tropics, Patancheru, India.

Name	Number of accessions	Ara h 1 (µg/g) Range and mean	Ara h 2 (µg/g) Range and mean	Ara h 3 (µg/g) Range and mean	Ara h 6 (µg/g) Range and mean	Ara h 8 (µg/g) Range and mean
*Variation in cultivated and wild accessions*						
Cultivated	264	4–36,833	52–77,042	22–106,765	829–103,892	0.01–70.12
		3,789	7,664	6,008	19,962	6
Wild	36	28–1,293	516–33,055	1,185–20,474	4,381–56,858	0.3–11
		404	606	6,857	13,420	2
*Variation between two subspecies*						
*A. hypogaea* ssp. *hypogaea*	96	29–22,000	560–62,350	93–63,720	3,264–57,504	0.5–20
		2,005	8,077	7,015	19,844	6
*A. hypogaea* ssp. *fastigiata*	148	4.0–36,833	41–57,915	22–106,765	829–103,892	0.1–38
		4,694	6,690	5,276	18,855	5
*Variation in agronomic type*						
Spanish bunch	72	4.0–8,267	41–57,915	22–20,574	3,022–63,867	0.01–70.12
		1,576	6,139	4,120	17,887	5
Valencia bunch	68	28–36,833	1,017–20,734	68–32,380	829–103,892	0.1–12
		8,295	7,084	5,241	19,581	5
Virginia bunch	53	30–12,470	560–22,695	104–63,720	5,311–57,504	0.5–19
		1,700	7,929	6,201	20,432	6
Virginia runner	33	91–4,594	791–62,350	337–42,508	7,037–46,240	0.7–20
		1,332	8,449	7,468	20,301	6
*Variation among accessions with the different biological status*						
Landraces	105	28–13,977	41–62,350	22–106,765	3,657–51,024	0.01–70.12
		2,272	7,630	7,970	19,251	6
Breeding material	52	60–36,833	1,874–25,900	131–323,801	829–103,892	0.1–12
		9,917	8,193	5,887	18,300	5
Improved cultivars	67	13–19,235	263–57,915	68–14,645	3,022–57,504	0.1–20
		1,915	7,187	3,038	20,183	5
Wild accessions	33	28–1,293	516–33,055	1,185–20,474	4,381–56,858	0.3–11
		404	606	6,857	13,420	2
*Variation among accessions from different geographical regions*						
South Asia (SA)	93	28–36,833	561–20,734	61–43,408	829–103,892	0.2–19
		7,121	6,216	6,420	17,993	5
Southeast Asia (SEA)	23	50–11,014	660–25,900	89–20,574	3,910–63,867	0.1–19
		2,283	6,653	5,561	18,285	7
West and Central Africa (WCA)	21	64–6,980	95.7–21,847	337–34,818	4,381–54,830	1.5–24
		1,962	9,130	10,595	17,098	7
East and Southern Africa (ESA)	30	60–13,977	1,017–62,350	105–16,132	6,395–47,041	0.9–70.12
		1,805	11,210	4,137	22,010	7
South America	72	28–6,727	516–33,055	68–63,720	3,657–56,858	0.3–15
		1,184	7,615	5,624	16,272	4
North America	27	12.5–12,470	262–22,696	98–9,431	5,756–57,504	0.1–20
		1,514	6,494	2,637	20,132	5
Remaining South America	19	288–7,867	2,812–15,267	42–106,765	5,586–42,552	0.1–11
		2,243	8,338	8,476	20,958	5

### Phenotypic Variation for Allergens Between Subspecies *hypogaea* and *fastigiata*

Phenotyping result showed a wide variation for all the five allergens between two subspecies of cultivated peanut i.e., *A. hypogaea* ssp. *hypogaea* (96 accessions) and *A. hypogaea* ssp. *fastigiata* (148 accessions). In ssp. *hypogaea*, the allergen content ranged from 29 to 22,000 µg/g for Ara h 1, 560 to 62,350 µg/g for Ara h 2, 93 to 63,720 µg/g for Ara h 3, 3,264 to 57,504 µg/g for Ara h 6, and 0.5 to 20 µg/g for Ara h 8 whereas in ssp. *fastigiata*, the allergen content varied from Ara h 1 (4–36,833 µg/g), Ara h 2 (41–57,915 µg/g), Ara h 3 (22–106,765 µg/g), Ara h 6 (829–103,892 µg/g), and Ara h 8 (0.3–11 µg/g) (**Table 1**). Excluding Ara h 1 (2,005 µg/g), the average amount of Ara h 2 (8,007 µg/g), Ara h 3 (7,015 µg/g), Ara h 6 (19,844 µg/g), and Ara h 8 (6 µg/g) was higher in ssp. *hypogaea* as compared to ssp. *fastigiata* i.e., Ara h 1 (4,694 µg/g), Ara h 2 (6,690 µg/g), Ara h 3 (5,276 µg/g), Ara h 6 (18,855 µg/g), and Ara h 8 (5 µg/g) (**Table 1** and **Figure 2B**). Even the average amount of allergens was detected high in ssp. *hypogaea*, the hypoallergen lines *i.e.*, ICG 1534 (4 µg/g) for Ara h 1, ICG 13491 (41 µg/g) for Ara h 2, ICG 6375 (22 µg/g) for Ara h 3, and ICGV 1328 (829 µg/g) for Ara h 6 were identified from ssp. *fastigiata* (**Table 1**). The above results were also supported by HCA. In HCA analysis, cluster 1 formed on the basis of the low quantity of allergens in their accessions. In cluster 1 the allergen content ranged from 12.5 to 27,375 µg/g for Ara h 1, 40.5 to 20,600 µg/g for Ara h 2, 55.43 to 23,306 µg/g for Aar h 3, 829 to 16,483 µg/g for Ara h 6, and 0.067 to 70 µg/g for Ara h 8 (**Supplementary Table 2** and **Figure 1B**).

### Phenotypic Variation for Allergens Among Different Peanut Agronomic Types

Phenotyping of accessions from different agronomic types namely Spanish bunch (72 accessions), Valencia bunch (68 accessions), Virginia bunch (53 accessions), and Virginia runner (33 accessions) indicated significant variation (**Table 1** and **Figure 2C**). Although the average amount of Ara h 1 was lowest in Virginia runner (1,332 µg/g) followed by Spanish bunch (1,576 µg/g), Virginia bunch (1,700 µg/g), and Valencia bunch (8,295 µg/g), however, the most hypoallergen line, ICG 1534 (4 µg/g) belongs to Spanish bunch (**Table 1** and **Figure 2C**). Likewise, Spanish bunch (17,887 µg/g) agronomic type had the lowest average for Ara h 6 as compared to other agronomic types but the most hypoallergen line was identified from Valencia bunch, ICGV 1328 (829 µg/g) (**Table 1**). Interestingly for Ara h 2 (6,139 µg/g), Ara h 3 (4,120 µg/g), and Ara h 8 (5 µg/g), the accessions with minimum average as well most hypoallergen line were detected in Spanish bunch agronomic type (**Table 1**).

### Phenotypic Variation for Allergens Among Peanut Accessions of Cultivated Gene Pool With Different Biological Status

The reference set included 105 traditional cultivars/landraces, 52 breeding/research material, 67 advanced/improved cultivars, and 36 wild accessions. Except for Ara h 3 (6,857 µg/g), the average amount of Ara h 1 (404 µg/g), Ara h 2 (606 µg/g), Ara h 6 (13,420 µg/g), and Ara h 8 (2 µg/g) was lower in wild accessions as compared to all the biological status groups of cultivated gene pool (**Table 1** and **Figure 2D**). Interestingly, most hypoallergen lines were detected in different biological status groups of cultivated gene pool. For example, Ara h 1 in advanced/improved cultivars (13–19,235 µg/g), Ara h 2 in traditional cultivars/landraces (41–62,350 µg/g), Ara h 6 in breeding/research material (829–103,892 µg/g), and Ara h 8 in traditional cultivars/landraces (0.01–70 µg/g). Similarly, the average amount of Ara h 3 allergens was found lowest in advanced/improved cultivars (3,038 µg/g) but the hypoallergen line was identified in traditional cultivars/landraces (41–62,350 µg/g) (**Table 1**).

### Phenotypic Variation for Allergens Among Peanut Accessions Representing Different Geographical Regions

**Figure 3:**
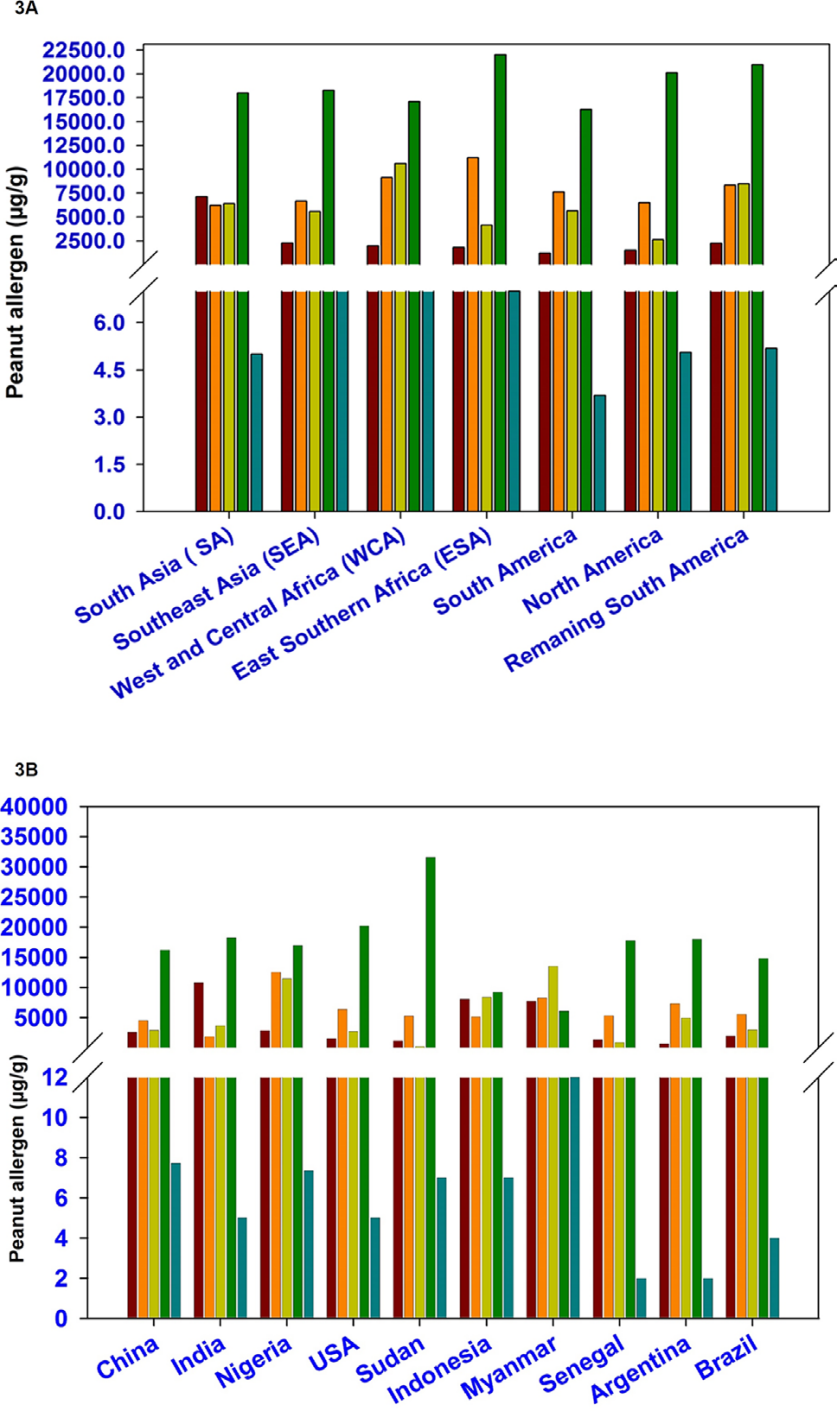
Phenotypic variation for five major peanut allergens among peanut germplasm lines representing various geographical regions **(3A)** and top 10 peanut producing countries **(3B)**.

The phenotyped set of 300 accessions represent different geographical regions namely South Asia (SA, 93 accessions), Southeast Asia (SEA, 23 accessions), West and Central Africa (WCA, 21 accessions), East and Southern Africa (ESA, 30 accessions), South America (72 accessions), North America (27 accessions), and 19 accessions from remaining South America. The average amount for Ara h 1 was lowest in accession representing South America (1,184 µg/g) followed by North America (1,514 µg/g), ESA (1,805 µg/g), WCA (1,962 µg/g), remaining South America (2,243 µg/g), SEA (2,283 µg/g), and SA (7,121). Likewise, the average amount of Ara h 2, Ara h 3, Ara h 6, and Ara h 8 were identified in SA (6,216 µg/g), North America (2,637 µg/), WCA (17,098), and South America (4 µg/g), respectively (**Table 1** and **Figure 3A**). The genotypes available in North America region especially in the USA had low amount of Ara h 1 (12.5–12,470 µg/g) as compared to genotypes grown in other parts of the world while the average content for Ara h 2 was low in WCA (41–21,847 µg/g) (**Table 1** and **Figure 3A**). The genotypes from South America had low amount of Ara h 3 (68–63,720 µg/g). In general, the allergen content for Ara h 6 was high and low for Ara h 8 across geographical regions. The most hypoallergen line for Ara h 6 (829 µg/g) was identified from the SA region (**Table 1** and **Figure 3A**). Cluster analysis also revealed that the North America region having low allergen lines for Ara h 1 (4–21,999 µg/g) and Ara h 2 (40.5–20,600 µg/g) while South America region having hypoallergen lines for Ara h 3 (53.43–23,306 µg/g) (**Supplementary Table 2** and **Figure 1B**).

### Phenotypic Variation for Allergens Among Peanut Accessions Representing Top 10 Peanut Producing Countries

Top 10 peanut producing countries include China, India, Nigeria, USA, Sudan, Indonesia, Myanmar, Senegal, Argentina, and Brazil which together contributed more than 81% of total global peanut production. Lowest average amount of Ara h 1 was found in Argentina (673 µg/g) followed by Sudan (1,174 µg/g), Senegal (1,383 µg/g), USA (1,554 µg/g), Brazil (1,968 µg/g), Nigeria (2,874 µg/g), Indonesia (4,037 µg/g), China (4,138 µg/g), Myanmar (7,714 µg/g), and India (**Table 2**; **Supplementary Table 3**). But the germplasm used in the USA had comparatively lower for Ara h 1 (13–5,023 µg/g) (**Table 2** and **Figure 3B**), followed by India (28–36,833 µg/g) and China (51–63,910 µg/g). Likewise for Ara h 2, USA (263–22,969 µg/g) (**Table 2** and **Figure 3B**) showed hypoallergen lines even the average content of Ara h 2 was lower in Indian germplasm line (1,876 µg/g). The hypoallergen lines for Ara h 3 (61–43,408 µg/g) and Ara h 6 (829–103,892 µg/g) were identified in Indian germplasm lines even though the average content of Ara h 3 (183 µg/g) and Ara h 6 (6,087 µg/g) was lower in Sudan and Myanmar germplasm lines. (**Table 2**, **Supplementary Table 3** and **Figure 3B**).

**Table 2:** Range and mean value for 5 major peanut allergens in peanut germplasm lines of the reference set representing top 10 peanut producing countries.

S. No	Country	Accession number	Ara h 1 (µg/g) Range and mean	Ara h 2 (µg/g) Range and mean	Ara h 3 (µg/g) Range and mean	Ara h 6 (µg/g) Range and mean	Ara h 8 (µg/g) Range and mean
1	China	10	51–6,391	660–6,331	89–11,897	5,196–39,660	0.1–19
			4,138	4,362	2,921	16,201	8
2	India	90	28–36,833	581–20,734	61–43,408	829–103,892	0.2–18.5
			10,796	1,876	3,672	18,285	5
3	Nigeria	5	799–5,023	8,146–21,847	841–19,819	6,467–25,401	0.1–20
			2,874	12,533	11,464	16,970	7
4	USA	26	13–1,250	263–22,696	98–9,431	5,756–57,504	0.1–20
			1,554	6,403	2,700	20,182	5
5	Sudan	3	599–2,109	3,984–6,618	105–225	19,825–47,041	3.6–8.7
			1,174	5,317	183	31,612	7
6	Indonesia	3	432–11,014	2,997–6,306	1,371–20,574	3,910–19,197	3–5.7
			4,037	4,807	9,357	9,247	7
7	Myanmar	2	1,614–13,813	7,211–9,371	7,197–19,902	6,087	12
			7,714	8,291	13,550	6,087	12
8	Senegal	2	65–2,611	2,998–7,673	337–1,341	13,541–22,023	1.6–3
			1,338	5,336	839	17,782	2
9	Argentina	22	78–4,177	516–15,757	131–20,456	4,381–56,850	0.3–8
			673	7,297	4,975	17,951	2
10	Brazil	15	49–6,727	842–17,544	68–7,542	6,874–27,561	0.4–11
			1,968	5,536	3,016	14,837	4

### Hypoallergen Lines for Five Major Peanut Allergens (Ara h 1, Ara h 2, Ara h 3, Ara h 6, and Ara h 8)

A wide variation among the 300 germplasm lines was observed for the presence of five major allergens i.e., Ara h 1 (4–36,833 µg/g), Ara h 2 (41–77,041 µg/g), Ara h 3 (22–106,765 µg/g), Ara h 6 (829–103,892 µg/g), and Ara h 8 (0.01–70.12 µg/g). Some germplasm lines showed low allergen content for combinations of two to three allergens.

**Figure 4:**
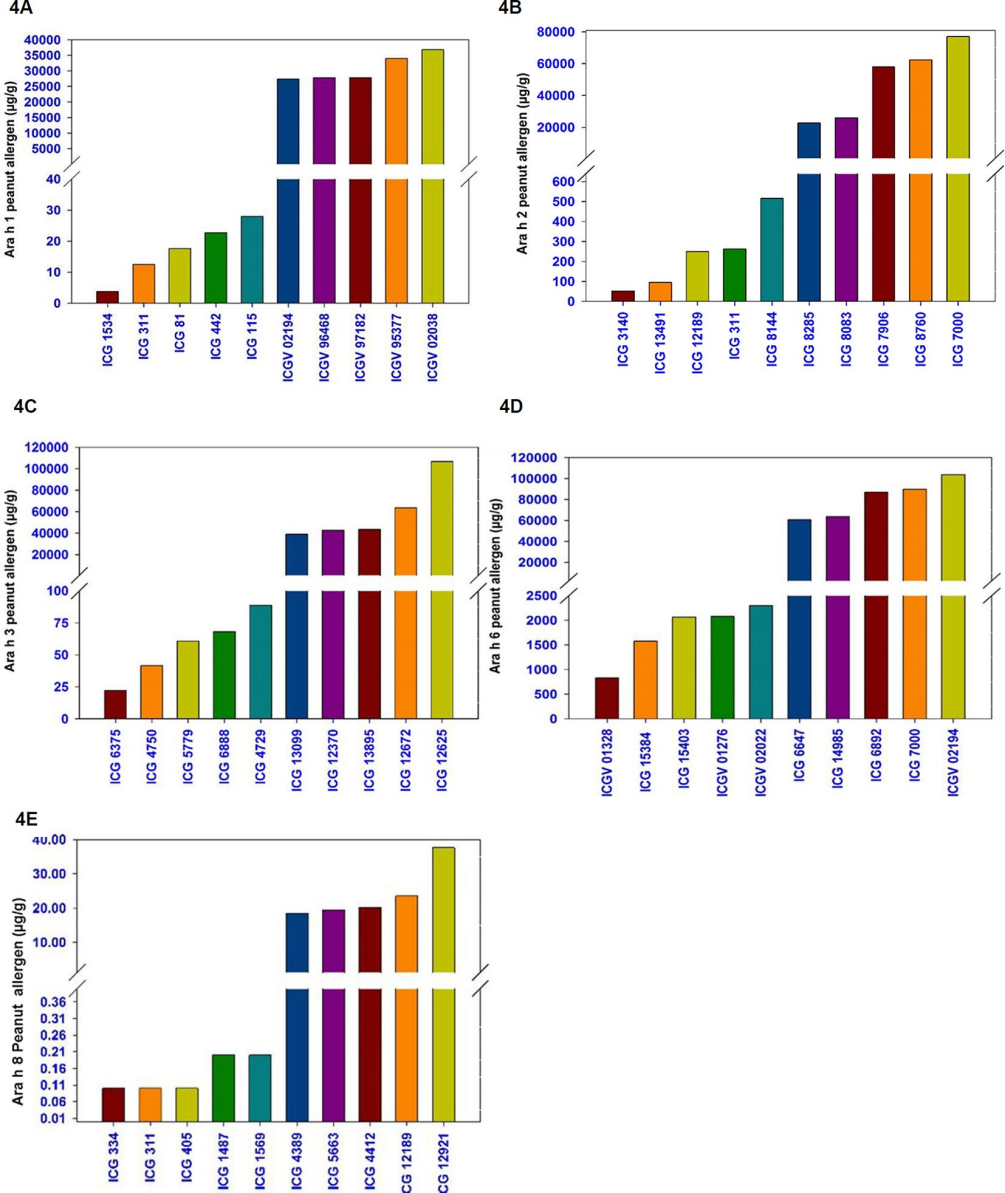
Selected germplasm lines with minimum and the maximum amount of allergen protein. **(4A)** Five peanut lines with highest and five lines with lowest allergen content for Ara h1 **(4B)** Ara h 2, **(4C)** Ara h 3, **(4D)** Ara h 6, and **(4E)** Ara h 8.

For Ara h 1, the phenotypic variation ranged from 4 µg/g (ICG 1534) to 36,833 µg/g (ICGV 02038) among cultivated accessions while it ranged from 28 µg/g (ICG 8124) to 1,293 µg/g (ICG 11555) among wild accessions (**Table 1** and **Figure 4A**). The most hypoallergen lines for Ara h 1 included ICG 1534 (4 µg/g) followed by ICG 311 (12.5 µg/g), ICG 81 (17.6 µg/g), ICG 442 (22.8 µg/g), and ICG 115 (27.9 µg/g) (**Table 3** and **Figure 4A**). All these hypoallergen lines belong to Spanish bunch types of *A. hypogaea* ssp. *fastigiata* except ICG 115 which belongs to Valencia bunch of the same subspecies (**Table 3** and **Figure 4A**). The best low allergen lines can be used for conducting further research on genomics and breeding.

**Table 3:** List of accessions having minimum and maximum allergen protein content for Ara h 1, Ara h 2, and Ara h 3 from peanut germplasm lines.

S. No.	Accessions	Species	Sub-species	Botanical type	Agronomic type	Peanut allergen content (µg/g)
*Low and high allergen lines for Ara h 1*						
1	ICG 1534	*A. hypogaea*	*fastigiata*	Vulgaris	Spanish bunch	4
2	ICG 311	*A. hypogaea*	*fastigiata*	Vulgaris	Spanish bunch	12.5
3	ICG 81	*A. hypogaea*	*fastigiata*	Vulgaris	Spanish bunch	17.6
4	ICG 442	*A. hypogaea*	*fastigiata*	Vulgaris	Spanish bunch	22.8
5	ICG 115	*A. hypogaea*	*fastigiata*	Fastigiata	Valencia bunch	27.9
6	ICGV 02194	*A. hypogaea*	*fastigiata*	Fastigiata	Valencia bunch	27,375
7	ICGV 96468	*A. hypogaea*	*fastigiata*	Fastigiata	Valencia bunch	27,831
8	ICGV 97182	*A. hypogaea*	*fastigiata*	Fastigiata	Valencia bunch	27,831
9	ICGV 95377	*A. hypogaea*	*fastigiata*	Fastigiata	Valencia bunch	34,034
10	ICGV 02038	*A. hypogaea*	*fastigiata*	Fastigiata	Valencia bunch	36,833
*Low and high allergen lines for Ara h 2*						
1	ICG 13491	*A. hypogaea*	*fastigiata*	Vulgaris	Spanish bunch	41.0
2	ICG 3140	*A. hypogaea*	*INA*	INA	INA	52.4
3	ICG 12189	*A. hypogaea*	*fastigiata*	Vulgaris	Spanish bunch	249
4	ICG 311	*A. hypogaea*	*fastigiata*	Vulgaris	Spanish bunch	262
5	ICG 8144	*A. villosa*	*INA*	Villosa	INA	516
6	ICG 8285	*A. hypogaea*	*hypogaea*	Hypogaea	Virginia bunch	22,695
7	ICG 8083	*A. hypogaea*	*fastigiata*	Vulgaris	Spanish bunch	25,899
8	ICG 7906	*A. hypogaea*	*fastigiata*	Vulgaris	Spanish bunch	57,915
9	ICG 8760	*A. hypogaea*	*hypogaea*	Hypogaea	Virginia runner	62,350
10	ICG 7000	*A. hypogaea*	*hypogaea*	Hypogaea	Virginia bunch	77,041
*Low and high allergen lines for Ara h 3*						
1	ICG 6375	*A. hypogaea*	*fastigiata*	Vulgaris	Spanish bunch	22
2	ICG 4750	*A. hypogaea*	*fastigiata*	Vulgaris	Spanish bunch	41
3	ICG 5779	*A. hypogaea*	*fastigiata*	Vulgaris	Spanish bunch	61
4	ICG 6888	*A. hypogaea*	*fastigiata*	Fastigiata	Valencia bunch	68
5	ICG 4729	*A. hypogaea*	*fastigiata*	Vulgaris	Spanish bunch	89
6	ICG 13099	*A. hypogaea*	*hypogaea*	Hypogaea	Virginia runner	38,988
7	ICG 12370	*A. hypogaea*	*hypogaea*	Hypogaea	Virginia runner	42,508
8	ICG 13895	*A. hypogaea*	*hypogaea*	Hypogaea	INA	43,408
9	ICG 12672	*A. hypogaea*	*hypogaea*	Hypogaea	Virginia bunch	63,720
10	ICG 12625	*A. hypogaea*	*fastigiata*	Aequitoriana	INA	106,765

S. No. 1–5 are accessions with low allergen (hypoallergen) protein content while 6–10 are accessions with high allergen protein content. INA, information not available.

A wide variation also observed in the presence of Ara h 2 and it ranged from 52 µg/g (ICG 3140) to 77,042 µg/g (ICGV 7000) among cultivated accessions (264) while it ranged from 516 µg/g (ICG 8144) to 15,757 µg/g (ICG 13218) among wild germplasm accessions (**Table 1** and **Figure 2A**). The most hypoallergen lines for Ara h 2 were ICG 13491 (41 µg/g), followed by ICG 3140 (52 µg/g), ICG 12189 (249 µg/g), ICG 311 (262 µg/g), and ICG 8144 (516 µg/g) (**Table 3** and **Figure 4B**). All these hypoallergen lines belonging to Spanish bunch types of *A. hypogaea* ssp. *fastigiata*. Interestingly ICG 311 belonging to Spanish bunch types of *A. hypogaea* ssp. *fastigiata* also had low Ara h 1 (12.5 µg/g) (**Table 3**).

The phenotypic variation of Ara h 3 in cultivated genotypes ranged from 22 µg/g (ICG 6375) to 106,765 µg/g (ICG 12625) while in wild accessions ranged from 1,185 µg/g (ICG 8135) to 20,474 µg/g (ICG 1156) (**Table 1**). The most hypoallergen lines for Ara h 3 included ICG 6375 (22 µg/g) followed by ICG 4750 (41 µg/g), ICG 15779 (61 µg/g), ICG 6888 (68 µg/g), and ICG 4729 (89 µg/g) (**Table 3** and **Figure 4C**). All these hypoallergen lines belong to Spanish bunch types of *A. hypogaea* ssp. *fastigiata* except ICG 6888 (68 µg/g) which belongs to Valencia bunch of the same subspecies (**Table 3** and **Figure 4C**).

For Ara h 6, the phenotypic wide variation identified in cultivated genotypes, and it ranged from 829 µg/g (ICGV 01328) to 103,892 µg/g (ICGV 02194) compared to wild accessions ranged from 4,381 µg/g (ICG 8123) to 56,857 µg/g (ICG 8195) (**Table 1**). The most hypoallergen lines for Ara h 6 identified in cultivated genotypes namely ICG 01328 (829 µg/g) followed by ICG 15384 (1,577 µg/g), ICG 15403 (2,066 µg/g), ICGV 01276 (2,078 µg/g), and ICGV 02022 (2,298 µg/g) (**Table 4** and **Figure 4D**). All these hypoallergen lines belong to Valencia bunch type of *A. hypogaea* ssp. *fastigiata*.

**Table 4:** List of accessions having minimum and maximum allergen protein content for Ara h 6 and Ara h 8 from peanut germplasm lines.

S. No.	Genotypes	Species	Sub-species	Botanical Type	Agronomic type	Peanut allergen content (µg/g)
*Low and high allergen lines for Ara h 6*						
1	ICGV 01328	*A. hypogaea*	*fastigiata*	Fastigiata	Valencia bunch	829
2	ICG 15384	*A. hypogaea*	*fastigiata*	Fastigiata	Valencia bunch	1,577
3	ICG 15403	*A. hypogaea*	*fastigiata*	Fastigiata	Valencia bunch	2,066
4	ICGV 01276	*A. hypogaea*	*fastigiata*	Fastigiata	Valencia bunch	2,078
5	ICGV 02022	*A. hypogaea*	*fastigiata*	Fastigiata	Valencia bunch	2,298
6	ICG 6647	*A. hypogaea*	*INA*	INA	INA	60,737
7	ICG 14985	*A. hypogaea*	*fastigiata*	Vulgaris	Spanish bunch	63,867
8	ICG 6892	*A. hypogaea*	*INA*	INA	INA	87,074
9	ICG 7000	*A. hypogaea*	*INA*	INA	INA	89,782
10	ICGV 02194	*A. hypogaea*	*fastigiata*	Fastigiata	Valencia bunch	103,892
*Low and high allergen lines for Ara h 8*						
1	ICG 334	*A. hypogaea*	*fastigiata*	Vulgaris	Spanish bunch	0.01
2	ICG 311	*A. hypogaea*	*fastigiata*	Vulgaris	Spanish bunch	0.1
3	ICG 405	*A. hypogaea*	*fastigiata*	Fastigiata	Valencia bunch	0.1
4	ICG 1487	*A. hypogaea*	*fastigiata*	Vulgaris	Spanish bunch	0.2
5	ICG 1569	*A. hypogaea*	*fastigiata*	Vulgaris	Spanish bunch	0.2
6	ICG 4389	*A. hypogaea*	*hypogaea*	Hypogaea	Virginia runner	18.5
7	ICG 5663	*A. hypogaea*	*hypogaea*	Hypogaea	Virginia bunch	19.4
8	ICG 4412	*A. hypogaea*	*hypogaea*	Hypogaea	Virginia runner	20.2
9	ICG 12189	*A. hypogaea*	*fastigiata*	Vulgaris	Spanish bunch	41.26
10	ICG 12921	*A. hypogaea*	*fastigiata*	Vulgaris	Spanish bunch	70.12

S. No. 1–5 are accessions with low allergen protein content while 6–10 are accessions with high allergen protein content.

The phenotypic variation for Ara h 8 was very narrow in cultivated accessions and it ranged from 0.01 µg/g (ICG 334) to 70 µg/g (ICG 12921) compared to wild accessions ranging from 0.3 µg/g (ICG 13206) to 11.27 µg/g (ICG 8973) (**Table 1**). The best low allergen lines for Ara h 8 included ICG 334 (.01 µg/g) followed by ICG 311(0.1 µg/g), ICG 405(0.1 µg/g), ICG 1487 (0.2 µg/g), and ICG 1569 (0.2 µg/g) (**Table 4** and **Figure 4D**). All these hypoallergen lines belong to Spanish bunch type of *A. hypogaea* ssp. *fastigiata*.

## Discussion

Peanut allergy is now a global health problem and so far no permanent solution is available to deal with this menace. More importantly, the peanut is consumed in the form of several peanut-based products, therefore, making the life of an allergic person more complicated and difficult. Hypoallergen lines provide an alternative approach to avoid these adverse reaction caused by IgE (Tscheppe and Breiteneder, 2017; Satitsuksanoa et al., 2018). The skin, the respiratory tract, and the gastrointestinal tract are allergic to the peanut and peanut-based product (Sicherer et al., 1998) and cute urticaria, acute vomiting, laryngeal oedema, hypotension, and dysrhythmia are the common symptoms (Bock et al., 2001; Sampson et al., 2017). Peanut-based allergy is very risky, and even the ingestion of trace amounts of peanut can cause life threats in minutes (Bock et al., 2001).

### The Stand-Alone Effort for Phenotyping Large-Scale, Diverse Germplasm Set for Major Peanut Allergens Using Most Sensitive Enzyme-Linked Immunosorbent Assay Protocol

**Figure 5:**
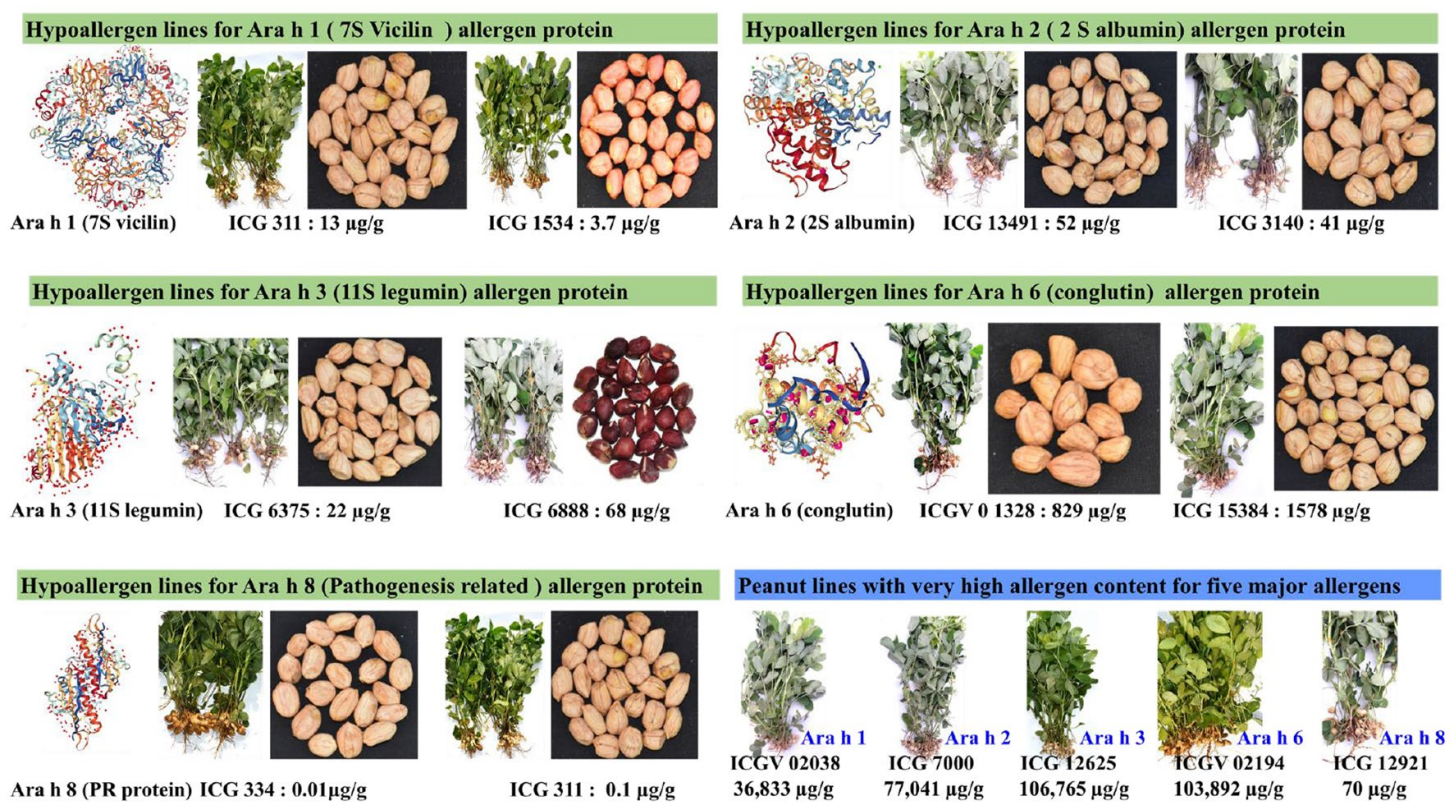
Plant and seed features of hypoallergen and high allergen peanut lines identified for five major peanut allergens (Ara h 1, Ara h 2, Ara h 3, Ara h 6, and Ara h 8).

Not much efforts have been done toward phenotyping a large peanut germplasm collections in the world. This is majorly due to lack of robust and high-throughput analytical assays to quantify major allergen proteins in peanut seeds. Recently our lab developed an ELISA based protocol to estimate major peanut allergens (Ara h 1, Ara h 2, Ara h 3, Ara h 6, and Ara h 8) using peanut seeds (Pandey et al., 2019). By using this protocol, we phenotyped 300 germplasm lines to quantify major peanut allergens. This study successfully identified hypoallergen lines for all the five allergens and this genetic variation for allergens can be exploited in crop improvement for developing improved hypoallergen lines (**Figure 5**). Using a pool of human serum from patients, a sample ELISA protocol was used to identify antigens in the peanut seed (Dodo et al., 2002) which reported no significant difference in the allergen content. Another such study on 53 Chinese peanut cultivars revealed that the allergenicity was caused by the allergen composition rather than a single allergen (Wu et al., 2016). This study also reported that the allergen content was high in all the peanut cultivars, however, the peanut allergen content could not be quantified in peanut seeds due to unavailability of antibodies. Hence our study is the first of its kind and identified a low/hypoallergen lines from peanut reference set to ensure food safety and security.

### Diverse Germplasm Set Representing 51 Countries Showed Wide Phenotypic Variation for Allergens

Results confirmed a wide variation of five major peanut allergens in the peanut reference set. This study used monoclonal antibodies for each allergen for phenotyping of ICRISAT reference set representing global diversity. These monoclonal antibodies also used to observe differences in specific peanut allergen profile in peanut flour and peanut-based products such as peanut butter, flour, and other confectionary preparations for clinical use (Filep et al., 2018). Screening of ICRISAT peanut reference set showed wide range of variation for all the five allergens i.e., Ara h 1 (4–36,833 µg/g), Ara h 2 (41–77,041 µg/g), Ara h 3 (22–106,765 µg/g), Ara h 6 (829–103,892 µg/g), and Ara h 8 (0.01–70 µg/g). Similar wide variation was also identified for Ara h 1, Ara h 2, and Ara h 3 in peanut butter, peanut powder, and peanut flour (Filep et al., 2018). An earlier study reported screening of 34 peanut accessions through patient sera, but no significant difference was observed for allergen content (Dodo et al., 2002). The other study also reported not much variation among 53 Chinese peanut cultivars (Wu et al., 2016) which may be due to the use of human sera to estimate the allergen content in their cultivars. These circumstances encouraged us to develop ELISA based protocol which can be used for quantifying allergen content in peanut kernels. Furthermore, we used the most diverse panel “reference set” consists of 300 genotypes which geographically represents 51 countries (Upadhyaya et al., 2003; Upadhyaya et al., 2010) and showed wide variation for all the five major allergens. The sensitivity of peanut allergens varied among populations in different geographical regions (Vereda et al., 2011). In USA and Sweden, the Ara h 1, Ara h 2, and Ara h 3 cause majority of the peanut allergenic reactions leading to serious illnesses. Similar trend has also been observed in 11 European countries (Ballmer-Weber and Beye, 2018). In contrast, the Spanish patients have less sensitivity to Ara h 1, Ara h 2, and Ara h 3 allergens and have shown more sensitivity to Ara h 9, lipid transfer protein. Similarly, the Spanish patients had the highest level of sensitivity rate to birch pollen allergen, Ara h 8, a cross-reactive homolog Bet v 1. It is important to note that despite few reports, not much have been reported from different Asian and African countries. The above difference in allergen sensitivity among countries and continents may have resulted due to several factors, including genetic makeup, environmental factors, and food habits.

### Hypoallergen Lines Identified for Major Peanut Allergens

Previous limited efforts in phenotyping closely related germplasm lines have not yielded in the identification of hypo-allergen peanut lines. Keeping in mind this fact, we explored a large number of diverse germplasm lines for phenotyping using newly developed very precise protocol (Pandey et al., 2019). As a result, this study reports low or hypoallergen lines for five major peanut allergens for the first time. For Ara h 1, five hypoallergen lines ranged between 4 and 28 µg/g and the ICG 1534 (4 µg/g) and ICGV 02038 (36,833 µg/g) had minimum Ara h 1 allergen protein. ICG 1534 belongs to Spanish bunch while ICGV 02038 to Valencia bunch. Similarly, the best hypoallergen lines for Ara h 2 were ICG 3140 with just 52.4 µg/g allergen protein. The accessions ICG 6375 (Spanish bunch), ICGV 01328 (Valencia Bunch), and ICG 334 (Spanish Bunch) were identified as best hypoallergen lines for Ara h 3, Ara h 6, and Ara h 8, respectively. The screening of 53 Chinese peanut cultivars through human sera, the Spanish bunch type having low peanut allergen content than the other agronomic type (Wu et al., 2016). They also reported that the Virginia type (Xinxiandahuasheng), Valencia type (Bangjihonghuasheng), Spanish type (Mangdou), and Peruvian type (Yaoshangxiaomake) are low allergen cultivars. Another study screened 35 US peanut cultivars using human antisera of the allergic patient but could not detect any significant variation (Dodo et al., 2002) which may be due to the narrow genetic base of these US cultivars derived from just two founder parents (Isleib and Wynne, 1992).

### Landraces Conserve Higher Diversity for Major Peanut Allergens

The landraces have shown less allergen protein accumulation for Ara h 1 (28–13,977 µg/g), Ara h 2 (41–62,350 µg/g), Ara h 3 (22–106,765 µg/g), Ara h 6 (3,657–51,024 µg/g), and Ara h 8 (0.01–70.12 µg/g) as compared to other biological groups i.e., breeding/research material, advanced/improved cultivar, and wild accessions. The quantification of five major allergens through immunological assay showed that the landraces conserved hypoallergen feature. These accessions are ICG 442 (22.7 µg/g) for Ara h 1, ICG 13491 (41 µg/g) for Ara h 2, ICG 6375 (22 µg/g) for Ara h 3, ICG 15405 (3,657 µg/g), and ICG 334 (0.01 µg/g). These accessions mostly belong to *fastigiata* subspecies and Spanish bunch types and can be used for developing hypoallergen lines through marker-assisted selection (MAS) or clusters of regularly interspaced short palindromic repeats (CRISPR)/Cas9 approach. One previous study reported that the landraces conserved genetic variation for edible oil properties and also suitable for biodiesel production in Algerian peanut landraces (Giuffre et al., 2016). This finding provides hope to use either directly cultivating or further improvement through breeding for developing hypoallergen lines. Some of the hypoallergen lines identified in this study have also been reported having resistance to multiple stresses, e.g., ICG 442, a Spanish hypoallergen line for Ara h 1 was reported resistant to multiple abiotic stresses such as drought, salinity, and phosphorus deficiency (Upadhyaya et al., 2014).

### A Sound Basis for Further Research and Cultivation of Hypoallergen Lines to Ensure Human Health From a Peanut Allergy

The development and release of several improved cultivars with high yield potential, biotic and abiotic stresses resistance, and enhanced/improved nutritional quality features in peanut has successfully been developed by combining the plant breeding techniques and efficient phenotyping methods. One of the previous studies reported that there are no significant differences in the allergen content among different peanut agronomic types consumed in western countries (Koppelman et al., 2016). However, that particular study involved very few numbers of genotypes representing various agronomic types. In our study, we used a large diverse peanut germplasm set and reporting that there are wide variation for allergen content among different agronomic types such as Spanish bunch, Valencia bunch, Virginia bunch, and Virginia runner. This study will provide hope to food industries to use hypoallergen lines in their food product preparations. Genetic improvement can be done using various modern tools and techniques through genomic research (Guo et al., 2012). Functional genomics and biotechnological techniques help discover and characterize agriculturaly important genes through deep analysis of the transcriptome, and their direct transfer to chosen cultivars (Brasileiro et al., 2014). Genes which encode storage protein, metabolic enzyme genes, genes involved in oil metabolism, and differentially expressed genes in response to pathogen stress, were identified and cloned in peanut by expressed sequence tag sequencing and are used to improve peanut production.

Wide varieties of peanut are grown to meet need of oil, food, and industries. The identified hypoallergen peanut lines can directly be used for cultivation and use in industry. Further, the identification of functional variation through genomics will facilitate the development of diagnostic markers for different allergens. The diagnostic markers can be used for improving varieties through MAS while the genes can be now edited through CRISPR/Cas9. CRISPR/Cas9 system has proven to be successful in various crop species over past years including wheat, tobacco, rice, potato, tomato sorghum, orange, and maize (Bortesi and Fischer, 2015). Although in peanut, there were no reports to implement genome editing, however, several reports of MAS and marker-assisted backcrossing (MABC) are available (Chu et al., 2011; Varshney et al., 2014; Janila et al., 2016; and Bera et al., 2018). CRISPR/Cas9 is able to introduce homozygous mutations into rice and tomato potentially accelerating crop improvement in the first generation of the transformants (Shen et al., 2014; Zhang et al., 2014). The elimination of allergen through genome editing technology would be useful for a specfiic group of customers. Silencing of Mal d 1 has decreased the allergenicity of apple, which may enhance the consumption without allergic reactions (Dubois et al., 2015). The immune dominant Ara h 2 peanut allergen successfully reduced the allergenicity in peanut through RNA interference technology (Dodo et al., 2008). All allergens coding genes should be silenced or removed in order to develop hypoallergen peanut that are safe for consumption by many patients, and the genome editing provide offers to do so effectively. The availability of hypoallergen lines will impact the peanut industry as well as contribute toward fighting the peanut allergy menace globally.

## Summary

The study identified several hypoallergen peanut lines for further study. These hypoallergen lines can be directly used for commercial cultivation in addition to further breeding research for developing improved peanut varieties by combining several other agronomic traits. The output of this study also encourages researchers to identify functional variation so that molecular breeding through MAS, MABC, and genome editing can be deployed for developing new hypoallergen lines in peanut. The results have shown great hope toward fighting peanut allergy and ensuring enhanced food safety and security for humans as well as promises good opportunity for economic gains by producers, processors, and industry.

## Data Availability Statement

All datasets generated for this study are included in the article/**Supplementary Material**.

## Author Contributions

MP conceived the idea. MP, AP, HS, HU, and RV designed the experiments. AP performed the experiment. AP and HS analyzed the Data. AP, RV, HS, and MP wrote the manuscript.

## Conflict of Interest

The authors declare that the research was conducted in the absence of any commercial or financial relationships that could be construed as a potential conflict of interest.
